# Supplementary material for: Spatial Localization of Recent Ancestors for Admixed Individuals
Source: G3 (Bethesda). 2014 Nov 3;4(12):2505–18. doi: 10.1534/g3.114.014274 (PMC4267945; doi:10.1534/g3.114.014274)
Supplement: Supporting Information [file supp_4_12_2505__index.html]

Spatial Localization of Recent Ancestors for Admixed Individuals — Supporting Information 

# Spatial Localization of Recent Ancestors for Admixed Individuals

## Supporting Information for Yang *et al.*, 2014

**Files in this Data Supplement:**

- Supporting Information - File S1, Figures S1-S3, Tables S1-S3, and References (PDF, 850 KB)
- File S1 - Supplementary Note (PDF, 137 KB)
- Figure S1 - Example of local ancestry prediction results for distant and close ancestors. (PDF, 585 KB)
- Figure S2 - Average Prediction error (Km) for six country pairs with largest populations. (PDF, 92 KB)
- Figure S3 - Number for simulations for six country pairs with largest populations. (PDF, 92 KB)
- Table S1 - Average distance between inferred and true ancestry locations in simulated admixed individuals from POPRES data. (PDF, 71 KB)
- Table S2 - Average distance between inferred and true ancestry locations in simulated admixed individuals from POPRES data.
- Table S3 - The outliers from SPAMIX analysis. (PDF, 57 KB)
