# Supplementary material for: Spatial Localization of Recent Ancestors for Admixed Individuals
Source: G3 (Bethesda). 2014 Nov 3;4(12):2505–18. doi: 10.1534/g3.114.014274 (PMC4267945; doi:10.1534/g3.114.014274)
Supplement: Supporting Information [file supp_g3.114.014274_014274SI.pdf]

G3 · Genes | Genomes | Genetics

Spatial Localization of Recent Ancestors for Admixed Individuals

Wen-Yun Yang[1], Alexander Platt[2], Charleston Wen-Kai Chiang[2], Eleazar Eskin[1,3,4], John Novembre[5], and Bogdan Pasaniuc[3,4,6]
[1]Department of Computer Science, UCLA
[2]Department of Ecology and Evolutionary Biology, UCLA
[3]Interdepartmental Program in Bioinformatics, UCLA
[4]Department of Human Genetics, UCLA
[5]Department of Human Genetics, University of Chicago
[6]Department of Pathology and Laboratory Medicine, Geffen School of Medicine at UCLA

Corresponding Author:
Bogdan Pasaniuc
Department of Pathology & Laboratory Medicine
Geffen School of Medicine at University of California, Los Angeles
10833 Le Conte Ave, CHS 33-365
Phone: 310-825-3291
Fax: 310-825-4846
Email: bpasaniuc@mednet.ucla.edu

DOI: 10.1534/g3.114.014274

File S1
Supplementary Note

Expectation Maximization algorithm for haploid spatial ancestral inference

We would like to infer M ancestral location for a given mixed individual haplotype. This can be achieved by maximizing the likelihood function with respect to X as follows

$$L(h; X, \Pi) = \sum_{Z} P(Z; \Pi) \prod_{i=1}^{L} P(h_i | z_i; X)$$

By treating X as parameters and Z as hidden variables, this maximization falls in exactly the procedure of EM algorithm.

E step. In short, the expectation step is similar to forward-backward algorithm in HMM, which calculates the posterior probability of hidden variables Z given current estimation of ancestral locations $X^{(t)}$.

$$P(z_i = j | h; X^{(t)}) = \frac{\alpha_i(j)\beta_i(j)}{\sum_j \alpha_L(j)}$$

where α and β can be calculated recursively

$$\begin{aligned}
\alpha_1(j) &= (1/M) P(h_1 | z_1 = j; X^{(t)}) \\
\alpha_i(j) &= \sum_{j'} \alpha_{i-1}(j') P(z_i = j | z_{i-1} = j') P(h_i | z_i = j; X^{(t)}) \\
\beta_L(j) &= 1 \\
\beta_i(j) &= \sum_{j'} P(z_{i+1} = j' | z_i = j) P(h_{i+1} | z_{i+1} = j'; X^{(t)}) \beta_{i+1}(j')
\end{aligned}$$

M step. The maximization step needs to alternatively optimize the Q functions in X and in Π. The first can be done as follows

$$\begin{aligned}
& Q(X; X^{(t)}, \Pi^{(t)}) \\
= & \sum_{Z} P(Z | h; X^{(t)}, \Pi^{(t)}) \ln \left(P(Z; \Pi) \prod_{i} P(h_i | z_i; X) \right) \\
= & \sum_{j} \left(\sum_{i} P(z_i = j | h; X^{(t)}, \Pi^{(t)}) \ln P(h_i | z_i = j; x_j) \right) + \text{const.} \\
= & \sum_{i,j} C_{ij} \ln P(h_i | z_i = j; x_j) + \text{const.} \\
= & \sum_{i,j} C_{ij} q_i(x_j) + \text{const.} \qquad (1)
\end{aligned}$$

where C_{ij} denotes the constant $P(z_i = j | h, X^{(t)}, \Pi^{(t)})$, and

$$q_i(x) = \begin{cases} -\ln(1 + \exp(a_i^T x + b_i)) & h_i = 0 \\ -\ln(1 + \exp(-a_i^T x - b_i)) & h_i = 1 \end{cases}$$

We use Newton's method to perform the maximization step, which is a widely used optimization technique. The gradient for the Q function in (1) can be computed as follows

$$\frac{\partial Q}{\partial x_j} = \sum_{i} C_{ij} \eta_i(x_j)$$

where

$$\eta_i(x_j) = \begin{cases} \dfrac{1}{1+\exp(-a_i^T x_j - b_i)}(-a_i)^T & h_i = 0 \\ \dfrac{1}{1+\exp(a_i^T x_j + b_i)}(a_i)^T & h_i = 1 \end{cases}$$

The Hessian matrix for the Q function in (1) can be obtained as follows

$$\frac{\partial^2 Q}{\partial x_j^2} = \sum_i C_{ij}\theta_i(x_j)$$

where

$$\theta_i(x_j) = \frac{1}{1+\exp(-a_i^T x_j - b_i)} \cdot \frac{1}{1+\exp(a_i^T x_j + b_i)} \cdot (-a_i a_i^T)$$

We also need the maximize the Q function in Π, which can be derived as follows

$$\begin{aligned} & Q(\Pi; X^{(t)}, \Pi^{(t)}) \\ = & \sum_Z P(Z|h; X^{(t)}, \Pi^{(t)}) \ln \left(P(Z;\Pi) \prod_i P(h_i|z_i; X) \right) \\ = & \sum_j \sum_k \left(\sum_i P(z_i = j, z_{i-1} = k|h; X^{(t)}, \Pi^{(t)}) \ln P(z_i = j|z_{i-1} = k; \Pi) \right) + \text{const.} \\ = & \sum_i \sum_j \left(\sum_{k \neq j} P(z_i = j, z_{i-1} = k|h; X^{(t)}, \Pi^{(t)}) \ln \pi_j + P(z_i = j, z_{i-1} = j|h; X^{(t)}, \Pi^{(t)}) \ln(1 - \tau_i(1-\pi_j)) \right) + \text{const.} \\ = & \sum_{i,j} D_{ij} \ln \pi_j + E_{ij} \ln(1 - \tau_i(1-\pi_j)) + \text{const.} \end{aligned} \qquad (2)$$

where D_{ij} and E_{ij} denote the constants as follows

$$\begin{aligned} D_{ij} &= \sum_{k \neq j} P(z_i = j, z_{i-1} = k|h; X^{(t)}, \Pi^{(t)}) \\ E_{ij} &= P(z_i = j, z_{i-1} = j|h; X^{(t)}, \Pi^{(t)}) \end{aligned}$$

We use Newton's method to perform the maximization step. The gradient for Q function in (2) can be computed as follows

$$\frac{\partial Q}{\partial \pi_j} = \sum_{ij} \frac{D_{ij}}{\pi_j} + \frac{E_{ij}\tau_i}{1 - \tau_i(1-\pi_j)}$$

The Hessian matrix for the Q function in in (2) can be computed as follows

$$\frac{\partial^2 Q}{\partial \pi_j^2} = \sum_{ij} -\frac{D_{ij}}{\pi_j^2} - \frac{E_{ij}\tau_i^2}{(1 - \tau_i(1-\pi_j))^2}$$

Expectation Maximization algorithm for diploid spatial ancestral inference

We would like to infer $M + N$ ancestral location for a given mixed individual genotype. This can be achieved by maximizing the likelihood function with respect to X and Y as follows

$$L(g; X, Y) = \sum_Z P(Z) \prod_{i=1}^{L} P(g_i|z_i^p, z_i^m; X, Y)$$

By treating X and Y as parameters and Z as hidden variables, this maximization falls in exactly the procedure of EM algorithm.

E step. In short, the expectation step is similar to forward-backward algorithm in HMM, which calculates the posterior probability of hidden variables Z given current estimation of ancestral locations $X^{(t)}$.

$$P(z_i^p = j, z_i^m = k | g; X^{(t)}) = \frac{\alpha_i(j,k)\beta_i(j,k)}{\sum_{j,k} \alpha_L(j,k)}$$

where α and β can be calculated recursively

$$\begin{aligned}
\alpha_1(j,k) &= 1/(MN) P(g_1 | z_1^p = j, z_1^m = k) \\
\alpha_i(j,k) &= \sum_{j',k'} \alpha_{i-1}(j',k') P(z_i^p = j | z_{i-1}^p = j') P(z_i^m = k | z_{i-1}^m = k') P(g_i | z_i^p = j, z_i^m = k) \\
\beta_L(j,k) &= 1 \\
\beta_i(j,k) &= \sum_{j',k'} P(z_{i+1}^p = j' | z_i^p = j) P(z_{i+1}^m = k' | z_i^m = k) P(g_{i+1} | z_{i+1}^p = j', z_{i+1}^m = k') \beta_{i+1}(j',k')
\end{aligned}$$

M step. The maximization step needs to optimize the Q functions in X, Y, Π and Ω. The Q function in X and Y can be done as follows

$$\begin{aligned}
& Q(X, Y; X^{(t)}, Y^{(t)}, \Pi^{(t)}, \Omega^{(t)}) \\
= & \sum_{Z^p, Z^m} P(Z^p, Z^m | g; X^{(t)}, Y^{(t)}, \Pi^{(t)}, \Omega^{(t)}) \ln \left(P(Z^p; \Pi^{(t)}) P(Z^m; \Omega^{(t)}) \prod_i P(g_i | z_i^p, z_i^m; X, Y) \right) \\
= & \sum_{j,k} \left(\sum_i P(z_i^p = j, z_i^m = k | g; X^{(t)}, Y^{(t)}, \Pi^{(t)}, \Omega^{(t)}) \ln P(g_i | z_i^p = j, z_i^m = k; x_j, y_k) \right) + \text{const.} \\
= & \sum_{i,j,k} C_{ijk} \ln P(g_i | z_i^p = j, z_i^m = k; x_j, y_k) + \text{const.} \\
= & \sum_{i,j,k} C_{ijk} q_i(x_j, y_k) + \text{const.} \qquad (3)
\end{aligned}$$

where C_{ijk} denotes the constant $P(z_i^p = j, z_i^m = k | g, X^{(t)}, Y^{(t)}, \Pi^{(t)}, \Omega^{(t)})$, and

$$q_i(x,y) = \begin{cases} -\ln(1+\exp(a_i^T x + b_i)) - \ln(1+\exp(a_i^T y + b_i)) & g_i = 0 \\ \ln \left(\begin{array}{c} \dfrac{1}{(1+\exp(a_i^T x + b_i))(1+\exp(-a_i^T y - b_i))} \\ + \dfrac{1}{(1+\exp(-a_i^T x - b_i))(1+\exp(a_i^T y + b_i))} \end{array} \right) & g_i = 1 \\ -\ln(1+\exp(-a_i^T x - b_i)) - \ln(1+\exp(-a_i^T y - b_i)) & g_i = 2 \end{cases}$$

This function is not concave in general, since the function corresponding to heterozygous genotype $g_i = 1$ is not concave. But we can still use convex optimization techniques to get a local optimal solution. In practice, we observe that the function is concave almost all the time. Thus, this proposed algorithm can well converge to an optimal solution.

Note that there is a subtle connection from the above EM algorithm to the parental location inference algorithm given previously [1]. For parental location inference, the hidden variables Z^p and Z^m would be fixed instead of random. Thus, the EM algorithm would be reduced to the algorithm given previously, which is equivalent to one M-step in the above EM algorithm.

The gradient for the Q function in (3) can be computed as follows

$$\frac{\partial Q}{\partial x_j} = \sum_{i,k} C_{ijk} \eta_{ik}(x_j, y_k)$$

where

$$\eta_{ik}(x_j, y_k) = \begin{cases} -p_{ij}a_i & g_i = 0 \\ \dfrac{(1-2m_{ik})(1-p_{ij})p_{ij}}{p_{ij}(1-m_{ik}) + m_{ik}(1-p_{ij})} \cdot a_i & g_i = 1 \\ (1-p_{ij})a_i & g_i = 2 \end{cases}$$

The variables p_{ij} and m_{ik} are shorthands for the ith allele frequencies for paternal ancestry j and maternal ancestry k defined as

$$\begin{aligned} p_{ij} &= \frac{1}{1+\exp(-a_i^T x_j - b_i)} \\ m_{ik} &= \frac{1}{1+\exp(-a_i^T y_k - b_i)} \end{aligned}$$

The Hessian for the Q function in (3) can be computed as follows

$$\frac{\partial^2 Q}{\partial x_j^2} = \sum_{i,k} C_{ijk}\theta_{ik}(x_j, y_k)$$

where

$$\theta_{ik}(x_j, y_k) = \begin{cases} (1-p_{ij})p_{ij}(-a_i a_i^T) & g_i = 0 \\ (1-2m_{ik})\dfrac{\dfrac{(1-m_{ik})p_{ij}}{1-p_{ij}} - \dfrac{m_{ik}(1-p_{ij})}{p_{ij}}}{\left(\dfrac{1-m_{ik}}{1-p_{ij}} + \dfrac{m_{ik}}{p_{ij}}\right)^2}(-a_i a_i^T) & g_i = 1 \\ (1-p_{ij})p_{ij}(-a_i a_i^T) & g_i = 2 \end{cases}$$

and

$$\frac{\partial^2 Q}{\partial x_j \partial y_k} = \sum_i I(g_i = 1)\left[\frac{m_{ik}(1-m_{ik})(1-2m_{ik})p_{ij}(1-p_{ij})(1-2p_{ij})}{[(1-m_{ik})p_{ij} + (1-p_{ij})m_{ik}]^2} + \frac{2m_{ik}(1-m_{ik})p_{ij}(1-p_{ij})}{(1-m_{ik})p_{ij} + (1-p_{ij})m_{ik}}\right](-a_i a_i^T)$$

The function $I(g_i = 1)$ is an indicator function, which is equal to 1 if $g_i = 1$, and equal to 0 otherwise.

We also need to maximize the Q function in Π and Ω, which can be derived as follows

$$\begin{aligned} & Q(\Pi; X^{(t)}, Y^{(t)}, \Pi^{(t)}, \Omega^{(t)}) && (4) \\ = & \sum_{Z^p, Z^m} P(Z^p, Z^m | g; X^{(t)}, Y^{(t)}, \Pi^{(t)}, \Omega^{(t)}) \ln\left(P(Z^p; \Pi)P(Z^m; \Omega)\prod_i P(g_i | z_i^p, z_i^m; X, Y)\right) \\ = & \sum_j \sum_k \left(\sum_i P(z_i^p = j, z_{i-1}^p = k | h; X^{(t)}, Y^{(t)}, \Pi^{(t)}, \Omega^{(t)}) \ln P(z_i^p = j | z_{i-1}^p = k; \Pi)\right) + \text{const.} \\ = & \sum_i \sum_j \Bigg(\sum_{k \neq j} P(z_i^p = j, z_{i-1}^p = k | g; X^{(t)}, Y^{(t)}, \Pi^{(t)}, \Omega^{(t)}) \ln \pi_j \\ & + P(z_i^p = j, z_{i-1}^p = j | g; X^{(t)}, Y^{(t)}, \Pi^{(t)}, \Omega^{(t)}) \ln(1 - \tau_i(1-\pi_j))\Bigg) + \text{const.} \\ = & \sum_{i,j} D_{ij} \ln \pi_j + E_{ij} \ln(1 - \tau_i(1-\pi_j)) && (5) \end{aligned}$$

where D_{ij} and E_{ij} denote the constants as follows

$$
\begin{aligned}
D_{ij} &= \sum_{k \neq j} P(z_i^p = j, z_{i-1}^p = k | g; X^{(t)}, Y^{(t)}, \Pi^{(t)}, \Omega^{(t)}) \\
E_{ij} &= P(z_i^p = j, z_{i-1}^p = j | g; X^{(t)}, Y^{(t)}, \Pi^{(t)}, \Omega^{(t)})
\end{aligned}
$$

We use Newton's method to perform the maximization step. The gradient for Q function in (5) can be computed as follows

$$
\frac{\partial Q}{\partial \pi_j} = \sum_{ij} \frac{D_{ij}}{\pi_j} + \frac{E_{ij}\tau_i}{1 - \tau_i(1 - \pi_j)}
$$

The Hessian matrix for the Q function in in (2) can be computed as follows

$$
\frac{\partial^2 Q}{\partial \pi_j^2} = \sum_{ij} -\frac{D_{ij}}{\pi_j^2} - \frac{E_{ij}\tau_i^2}{(1 - \tau_i(1 - \pi_j))^2}
$$

Similarly, the derivation of Q function in Ω can be done by replacing all Z^p variables with Z^m.

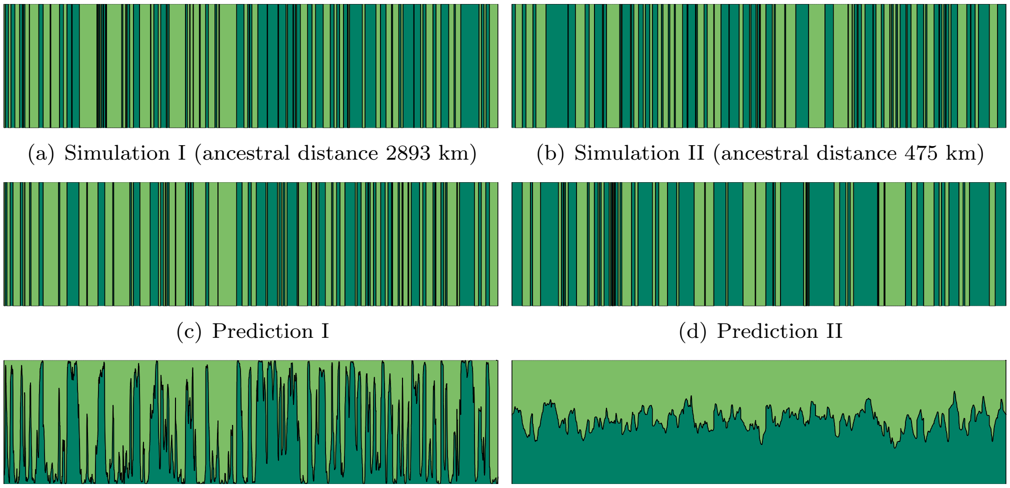

(a) Simulation I (ancestral distance 2893 km) (b) Simulation II (ancestral distance 475 km)

(c) Prediction I (d) Prediction II

(e) Posterior I (f) Posterior II

Figure S1: Example of local ancestry prediction results for distant and close ancestors.

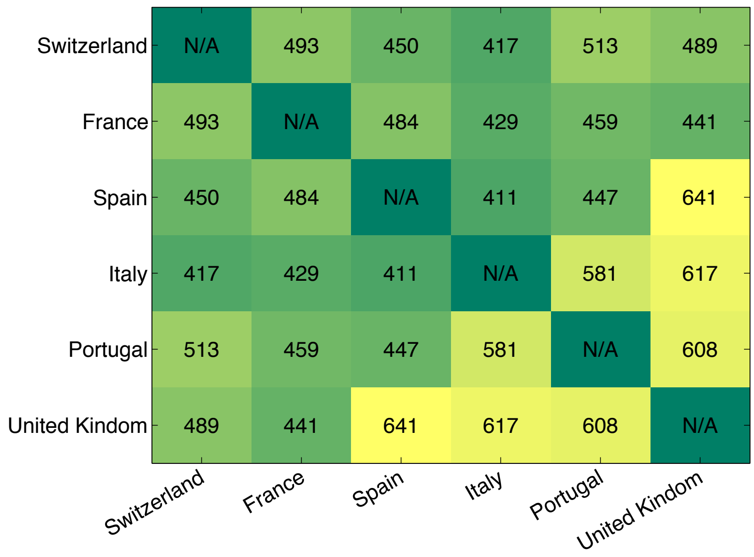

Figure S2: Average Prediction error (Km) for six country pairs with largest populations.

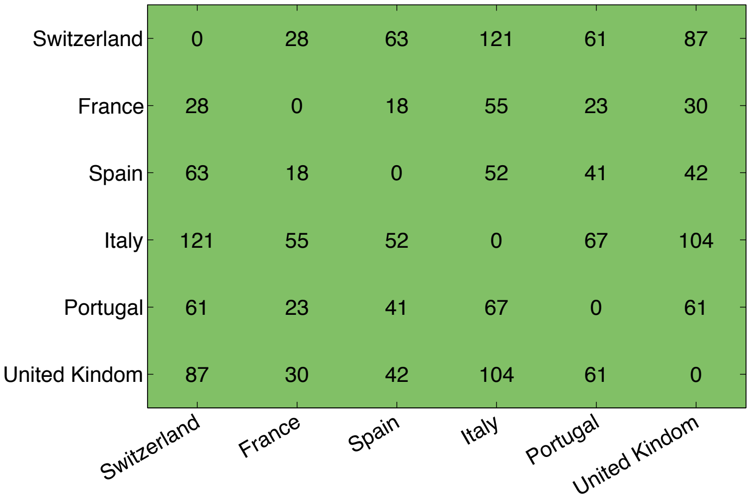

Figure S3: Number for simulations for six country pairs with largest populations.

Table S1: Average distance between inferred and true ancestry locations in simulated admixed individuals from POPRES data. Simulations assume 4 generations in the mixture process. Independent SNP model denotes the extension of SPA that ignores admixture-LD. It can also be understood as SPAMIX with completely random transition probability between nearby SNPs. SPAMIX (logistic) represents simulation results starting from haplotypes generated at a location on a map using a Bernoulli sampling from the logistic gradients (see Methods). Parenthesis denotes the standard deviations. We found that Linkage Disequilibrium (LD) significantly affects the ancestry inference as well as the local ancestry inference. We observe more recombination events than expected if using the correct recombination probability (used in simulations). We circumvent this bias multiplying the transition probability by a factor 10^{-1}, 10^{-2}, 10^{-4} and 10^{-5} for the pruned SNP list with 0.1, 0.2, 0.5 and 0.8 pruning thresholds. 44,699, 72,418, 136,284, 194,432 SNPs were retained at the 4 pruning thresholds.

No. of ancestry		1	2	3	4
Independent SNP model	Pruned SNP (0.1)	425(252)	961(540)	977(599)	982(655)
	Pruned SNP (0.2)	443(265)	880(491)	898(530)	880(578)
	Pruned SNP (0.5)	420(245)	823(448)	855(502)	810(494)
	Pruned SNP (0.8)	421(259)	810(429)	845(491)	813(505)
SPAMIX	Pruned SNP (0.1)	425(252)	558(314)	596(353)	621(405)
	Pruned SNP (0.2)	443(265)	550(326)	591(367)	639(423)
	Pruned SNP (0.5)	420(245)	557(359)	630(522)	657(617)
	Pruned SNP (0.8)	421(259)	589(557)	809(895)	878(848)

Table S2: Average distance between inferred and true ancestry locations in simulated admixed individuals from POPRES data. Independent SNP model denotes the extension of SPA that ignores admixture-LD. It can also be understood as SPAMIX with completely random transition probability between nearby SNPs. SPAMIX (logistic) represents simulation results starting from haplotypes generated at a location on a map using a Bernoulli sampling from the logistic gradients (see Methods). Parenthesis denote the standard deviations. We found that Linkage Disequilibrium (LD) significantly affects the ancestry inference as well as the local ancestry inference in unaccounted for. We observe more recombination events than expected if using the correct recombination probability (used in simulations). We circumvent this bias multiplying the transition probability by a factor 10^{-1}, 10^{-2}, 10^{-4} and 10^{-5} for the pruned SNP list with 0.1, 0.2, 0.5 and 0.8 pruning thresholds. 44,699, 72,418, 136,284, 194,432 SNPs were retained at the 4 pruning thresholds.

No. of generation		2	4	6	8
Independent SNP model	Pruned SNP (0.1)	995(550)	961(540)	974(539)	987(537)
	Pruned SNP (0.2)	899(487)	880(491)	864(466)	927(491)
	Pruned SNP (0.5)	809(444)	823(448)	819(436)	837(444)
	Pruned SNP (0.8)	834(441)	810(429)	812(442)	799(447)
SPAMIX	Pruned SNP (0.1)	549(318)	558(314)	567(334)	546(326)
	Pruned SNP (0.2)	548(329)	550(326)	541(295)	562(336)
	Pruned SNP (0.5)	551(390)	557(359)	590(371)	588(467)
	Pruned SNP (0.8)	580(478)	589(557)	634(576)	586(538)

Table S3: The outliers from SPAMIX analysis. They are POPRES admixed individuals with ancestral predictions inconsistent with their self-reported ancestries.

POPRES ID	self	father	PGF	PGM	mother	MGF	MGM	Pred. Locations
4183	Austria	Austria	Austria	Czech Republic	Poland	Russia	Switzerland	(52.26 12.61),(43.78 -9.81),(41.63 16.61),(52.44 13.30)
28710	France	Poland	Germany	Poland	France	France	France	(39.47 5.82),(37.97 14.65),(40.99 15.31)
24943	Sweden	Sweden	Sweden	Sweden	Finland	Russia	France	(48.03 6.91),(48.09 6.67),(57.35 8.79)
20086	France	France	France	France	France	Switzerland	France	(35.62 13.04),(43.65 16.14)
5550	Germany	Switzerland	Switzerland	Switzerland	Germany	Germany	Germany	(46.45 23.86),(55.66 -7.40)
47799	Germany	Germany	Russia	Germany	Germany	Germany	Germany	(50.20 14.77),(53.02 3.44)
32002	France	France	France	France	Turkey	Turkey	France	(35.85 10.05),(40.75 13.59)
27995	Poland	Poland	Poland	Poland	Russia	Russia	Poland	(55.65 12.49),(47.67 10.56)
38489	Russia	Russia	Germany	Germany	Switzerland	Switzerland	Russia	(46.59 5.62),(46.67 7.38),(49.86 -0.22)
7251	Austria	Switzerland	Switzerland	Switzerland	Austria	Austria	Austria	(38.64 -6.89),(62.19 30.99)
17323	Switzerland	Russia	Russia	Russia	Switzerland	Switzerland	Switzerland	(50.99 -1.41),(46.35 10.78)
20046	France	Russia	Russia	Russia	Poland	Poland	Poland	(37.25 14.09),(44.40 9.91)
24429	France	Russia	Russia	Russia	France	France	France	(50.59 1.93),(44.02 4.97)
39106	Israel	Greece	Greece	Greece	Russia	Germany	Sweden	(41.68 4.76),(36.35 23.47),(41.78 5.04)
49793	France	Romania	Romania	Romania	Russia	Russia	Russia	(41.31 9.54),(40.83 19.25)
47137	France	France	Germany	Germany	Austria	Switzerland	Switzerland	(38.01 12.40),(41.96 12.49)
34848	Egypt	Turkey	Turkey	Turkey	France	France	France	(38.75 9.47),(37.47 14.32)
10635	France	France	France	France	Russia	Russia	Bulgaria	(53.28 5.54),(45.50 5.56),(50.11 7.61)
18548	Czech Republic	Germany	Germany	Germany	Russia	Russia	Russia	(42.54 13.24),(38.67 9.20)
22423	Russia	Ukraine	Ukraine	Ukraine	Russia	Russia	Russia	(53.32 7.55),(50.79 17.17)
13411	France	Russia	Russia	Russia	France	France	France	(39.22 9.31),(46.77 5.19)
42867	Switzerland	Switzerland	Switzerland	Russia	Switzerland	Switzerland	Switzerland	(47.42 16.49),(49.47 -4.12)
33744	Switzerland	Switzerland	Russia	Germany	Spain	Switzerland	Switzerland	(42.39 13.98),(40.81 5.44),(50.07 2.19)
31350	Israel	Romania	Romania	Romania	Russia	Russia	Russia	(42.23 7.63),(38.30 19.55)
15990	France	Russia	Russia	Russia	Greece	Greece	Greece	(38.60 5.52),(38.88 15.46)

References

[1] Yang, W., Novembre, J., Eskin, E., and Halperin, E. (2012). A model-based approach for analysis of spatial structure in genetic data. Nature genetics *44*, 725–731.
