# Supplementary material for: Spatial Localization of Recent Ancestors for Admixed Individuals
Source: G3 (Bethesda). 2014 Nov 3;4(12):2505–18. doi: 10.1534/g3.114.014274 (PMC4267945; doi:10.1534/g3.114.014274)
Supplement: Supporting Information [file supp_g3.114.014274_FileS1.pdf]

File S1
Supplementary Note

Expectation Maximization algorithm for haploid spatial ancestral inference

We would like to infer M ancestral location for a given mixed individual haplotype. This can be achieved by maximizing the likelihood function with respect to X as follows

$$L(h; X, \Pi) = \sum_{Z} P(Z; \Pi) \prod_{i=1}^{L} P(h_i | z_i; X)$$

By treating X as parameters and Z as hidden variables, this maximization falls in exactly the procedure of EM algorithm.

E step. In short, the expectation step is similar to forward-backward algorithm in HMM, which calculates the posterior probability of hidden variables Z given current estimation of ancestral locations $X^{(t)}$.

$$P(z_i = j | h; X^{(t)}) = \frac{\alpha_i(j)\beta_i(j)}{\sum_j \alpha_L(j)}$$

where α and β can be calculated recursively

$$\begin{aligned}
\alpha_1(j) &= (1/M) P(h_1 | z_1 = j; X^{(t)}) \\
\alpha_i(j) &= \sum_{j'} \alpha_{i-1}(j') P(z_i = j | z_{i-1} = j') P(h_i | z_i = j; X^{(t)}) \\
\beta_L(j) &= 1 \\
\beta_i(j) &= \sum_{j'} P(z_{i+1} = j' | z_i = j) P(h_{i+1} | z_{i+1} = j'; X^{(t)}) \beta_{i+1}(j')
\end{aligned}$$

M step. The maximization step needs to alternatively optimize the Q functions in X and in Π. The first can be done as follows

$$\begin{aligned}
& Q(X; X^{(t)}, \Pi^{(t)}) \\
=& \sum_{Z} P(Z | h; X^{(t)}, \Pi^{(t)}) \ln \left(P(Z; \Pi) \prod_{i} P(h_i | z_i; X) \right) \\
=& \sum_{j} \left(\sum_{i} P(z_i = j | h; X^{(t)}, \Pi^{(t)}) \ln P(h_i | z_i = j; x_j) \right) + \text{const.} \\
=& \sum_{i,j} C_{ij} \ln P(h_i | z_i = j; x_j) + \text{const.} \\
=& \sum_{i,j} C_{ij} q_i(x_j) + \text{const.} \qquad (1)
\end{aligned}$$

where C_{ij} denotes the constant $P(z_i = j | h, X^{(t)}, \Pi^{(t)})$, and

$$q_i(x) = \begin{cases} -\ln(1 + \exp(a_i^T x + b_i)) & h_i = 0 \\ -\ln(1 + \exp(-a_i^T x - b_i)) & h_i = 1 \end{cases}$$

We use Newton's method to perform the maximization step, which is a widely used optimization technique. The gradient for the Q function in (1) can be computed as follows

$$\frac{\partial Q}{\partial x_j} = \sum_{i} C_{ij} \eta_i(x_j)$$

where

$$\eta_i(x_j) = \begin{cases} \frac{1}{1+\exp(-a_i^T x_j - b_i)}(-a_i)^T & h_i = 0 \\ \frac{1}{1+\exp(a_i^T x_j + b_i)}(a_i)^T & h_i = 1 \end{cases}$$

The Hessian matrix for the Q function in (1) can be obtained as follows

$$\frac{\partial^2 Q}{\partial x_j^2} = \sum_i C_{ij}\theta_i(x_j)$$

where

$$\theta_i(x_j) = \frac{1}{1+\exp(-a_i^T x_j - b_i)} \cdot \frac{1}{1+\exp(a_i^T x_j + b_i)} \cdot (-a_i a_i^T)$$

We also need the maximize the Q function in Π, which can be derived as follows

$$\begin{aligned} & Q(\Pi; X^{(t)}, \Pi^{(t)}) \\ = & \sum_Z P(Z|h; X^{(t)}, \Pi^{(t)}) \ln\left(P(Z;\Pi)\prod_i P(h_i|z_i;X)\right) \\ = & \sum_j\sum_k\left(\sum_i P(z_i=j, z_{i-1}=k|h; X^{(t)}, \Pi^{(t)}) \ln P(z_i=j|z_{i-1}=k;\Pi)\right) + \text{const.} \\ = & \sum_i\sum_j\left(\sum_{k\neq j} P(z_i=j, z_{i-1}=k|h; X^{(t)}, \Pi^{(t)}) \ln \pi_j + P(z_i=j, z_{i-1}=j|h; X^{(t)}, \Pi^{(t)}) \ln(1-\tau_i(1-\pi_j))\right) + \text{const.} \\ = & \sum_{i,j} D_{ij}\ln\pi_j + E_{ij}\ln(1-\tau_i(1-\pi_j)) + \text{const.} \end{aligned} \quad (2)$$

where D_{ij} and E_{ij} denote the constants as follows

$$\begin{aligned} D_{ij} &= \sum_{k\neq j} P(z_i=j, z_{i-1}=k|h; X^{(t)}, \Pi^{(t)}) \\ E_{ij} &= P(z_i=j, z_{i-1}=j|h; X^{(t)}, \Pi^{(t)}) \end{aligned}$$

We use Newton's method to perform the maximization step. The gradient for Q function in (2) can be computed as follows

$$\frac{\partial Q}{\partial \pi_j} = \sum_{ij}\frac{D_{ij}}{\pi_j} + \frac{E_{ij}\tau_i}{1-\tau_i(1-\pi_j)}$$

The Hessian matrix for the Q function in in (2) can be computed as follows

$$\frac{\partial^2 Q}{\partial \pi_j^2} = \sum_{ij} -\frac{D_{ij}}{\pi_j^2} - \frac{E_{ij}\tau_i^2}{(1-\tau_i(1-\pi_j))^2}$$

Expectation Maximization algorithm for diploid spatial ancestral inference

We would like to infer $M+N$ ancestral location for a given mixed individual genotype. This can be achieved by maximizing the likelihood function with respect to X and Y as follows

$$L(g; X, Y) = \sum_Z P(Z)\prod_{i=1}^{L} P(g_i|z_i^p, z_i^m; X, Y)$$

By treating X and Y as parameters and Z as hidden variables, this maximization falls in exactly the procedure of EM algorithm.

E step. In short, the expectation step is similar to forward-backward algorithm in HMM, which calculates the posterior probability of hidden variables Z given current estimation of ancestral locations $X^{(t)}$.

$$P(z_i^p = j, z_i^m = k|g; X^{(t)}) = \frac{\alpha_i(j,k)\beta_i(j,k)}{\sum_{j,k} \alpha_L(j,k)}$$

where α and β can be calculated recursively

$$\begin{aligned}
\alpha_1(j,k) &= 1/(MN)P(g_1|z_1^p = j, z_1^m = k) \\
\alpha_i(j,k) &= \sum_{j',k'} \alpha_{i-1}(j',k')P(z_i^p = j|z_{i-1}^p = j')P(z_i^m = k|z_{i-1}^m = k')P(g_i|z_i^p = j, z_i^m = k) \\
\beta_L(j,k) &= 1 \\
\beta_i(j,k) &= \sum_{j',k'} P(z_{i+1}^p = j'|z_i^p = j)P(z_{i+1}^m = k'|z_i^m = k)P(g_{i+1}|z_{i+1}^p = j', z_{i+1}^m = k')\beta_{i+1}(j',k')
\end{aligned}$$

M step. The maximization step needs to optimize the Q functions in X, Y, Π and Ω. The Q function in X and Y can be done as follows

$$\begin{aligned}
& Q(X,Y;X^{(t)},Y^{(t)},\Pi^{(t)},\Omega^{(t)}) \\
=& \sum_{Z^p,Z^m} P(Z^p,Z^m|g;X^{(t)},Y^{(t)},\Pi^{(t)},\Omega^{(t)}) \ln\left(P(Z^p;\Pi^{(t)})P(Z^m;\Omega^{(t)})\prod_i P(g_i|z_i^p,z_i^m;X,Y)\right) \\
=& \sum_{j,k}\left(\sum_i P(z_i^p = j, z_i^m = k|g;X^{(t)},Y^{(t)},\Pi^{(t)},\Omega^{(t)}) \ln P(g_i|z_i^p = j, z_i^m = k; x_j, y_k)\right) + \text{const.} \\
=& \sum_{i,j,k} C_{ijk} \ln P(g_i|z_i^p = j, z_i^m = k; x_j, y_k) + \text{const.} \\
=& \sum_{i,j,k} C_{ijk} q_i(x_j, y_k) + \text{const.} \qquad (3)
\end{aligned}$$

where C_{ijk} denotes the constant $P(z_i^p = j, z_i^m = k|g, X^{(t)}, Y^{(t)}, \Pi^{(t)}, \Omega^{(t)})$, and

$$q_i(x,y) = \begin{cases} -\ln(1+\exp(a_i^T x + b_i)) - \ln(1+\exp(a_i^T y + b_i)) & g_i = 0 \\ \ln\left(\begin{array}{c} \frac{1}{(1+\exp(a_i^T x + b_i))(1+\exp(-a_i^T y - b_i))} \\ +\frac{1}{(1+\exp(-a_i^T x - b_i))(1+\exp(a_i^T y + b_i))} \end{array}\right) & g_i = 1 \\ -\ln(1+\exp(-a_i^T x - b_i)) - \ln(1+\exp(-a_i^T y - b_i)) & g_i = 2 \end{cases}$$

This function is not concave in general, since the function corresponding to heterozygous genotype $g_i = 1$ is not concave. But we can still use convex optimization techniques to get a local optimal solution. In practice, we observe that the function is concave almost all the time. Thus, this proposed algorithm can well converge to an optimal solution.

Note that there is a subtle connection from the above EM algorithm to the parental location inference algorithm given previously [1]. For parental location inference, the hidden variables Z^p and Z^m would be fixed instead of random. Thus, the EM algorithm would be reduced to the algorithm given previously, which is equivalent to one M-step in the above EM algorithm.

The gradient for the Q function in (3) can be computed as follows

$$\frac{\partial Q}{\partial x_j} = \sum_{i,k} C_{ijk}\eta_{ik}(x_j, y_k)$$

where

$$\eta_{ik}(x_j, y_k) = \begin{cases} -p_{ij}a_i & g_i = 0 \\ \dfrac{(1-2m_{ik})(1-p_{ij})p_{ij}}{p_{ij}(1-m_{ik}) + m_{ik}(1-p_{ij})} \cdot a_i & g_i = 1 \\ (1-p_{ij})a_i & g_i = 2 \end{cases}$$

The variables p_{ij} and m_{ik} are shorthands for the ith allele frequencies for paternal ancestry j and maternal ancestry k defined as

$$\begin{aligned} p_{ij} &= \frac{1}{1+\exp(-a_i^T x_j - b_i)} \\ m_{ik} &= \frac{1}{1+\exp(-a_i^T y_k - b_i)} \end{aligned}$$

The Hessian for the Q function in (3) can be computed as follows

$$\frac{\partial^2 Q}{\partial x_j^2} = \sum_{i,k} C_{ijk}\theta_{ik}(x_j, y_k)$$

where

$$\theta_{ik}(x_j, y_k) = \begin{cases} (1-p_{ij})p_{ij}(-a_i a_i^T) & g_i = 0 \\ (1-2m_{ik})\dfrac{\dfrac{(1-m_{ik})p_{ij}}{1-p_{ij}} - \dfrac{m_{ik}(1-p_{ij})}{p_{ij}}}{\left(\dfrac{1-m_{ik}}{1-p_{ij}} + \dfrac{m_{ik}}{p_{ij}}\right)^2}(-a_i a_i^T) & g_i = 1 \\ (1-p_{ij})p_{ij}(-a_i a_i^T) & g_i = 2 \end{cases}$$

and

$$\frac{\partial^2 Q}{\partial x_j \partial y_k} = \sum_i I(g_i = 1)\left[\frac{m_{ik}(1-m_{ik})(1-2m_{ik})p_{ij}(1-p_{ij})(1-2p_{ij})}{[(1-m_{ik})p_{ij} + (1-p_{ij})m_{ik}]^2} + \frac{2m_{ik}(1-m_{ik})p_{ij}(1-p_{ij})}{(1-m_{ik})p_{ij} + (1-p_{ij})m_{ik}}\right](-a_i a_i^T)$$

The function $I(g_i = 1)$ is an indicator function, which is equal to 1 if $g_i = 1$, and equal to 0 otherwise.

We also need to maximize the Q function in Π and Ω, which can be derived as follows

$$\begin{aligned} & Q(\Pi; X^{(t)}, Y^{(t)}, \Pi^{(t)}, \Omega^{(t)}) \qquad (4) \\ = & \sum_{Z^p, Z^m} P(Z^p, Z^m | g; X^{(t)}, Y^{(t)}, \Pi^{(t)}, \Omega^{(t)}) \ln\left(P(Z^p; \Pi)P(Z^m; \Omega)\prod_i P(g_i | z_i^p, z_i^m; X, Y)\right) \\ = & \sum_j \sum_k \left(\sum_i P(z_i^p = j, z_{i-1}^p = k | h; X^{(t)}, Y^{(t)}, \Pi^{(t)}, \Omega^{(t)}) \ln P(z_i^p = j | z_{i-1}^p = k; \Pi)\right) + \text{const.} \\ = & \sum_i \sum_j \left(\sum_{k \neq j} P(z_i^p = j, z_{i-1}^p = k | g; X^{(t)}, Y^{(t)}, \Pi^{(t)}, \Omega^{(t)}) \ln \pi_j \right. \\ & \left. + P(z_i^p = j, z_{i-1}^p = j | g; X^{(t)}, Y^{(t)}, \Pi^{(t)}, \Omega^{(t)}) \ln(1 - \tau_i(1-\pi_j))\right) + \text{const.} \\ = & \sum_{i,j} D_{ij} \ln \pi_j + E_{ij} \ln(1 - \tau_i(1-\pi_j)) \qquad (5) \end{aligned}$$

where D_{ij} and E_{ij} denote the constants as follows

$$\begin{aligned} D_{ij} &= \sum_{k \neq j} P(z_i^p = j, z_{i-1}^p = k | g; X^{(t)}, Y^{(t)}, \Pi^{(t)}, \Omega^{(t)}) \\ E_{ij} &= P(z_i^p = j, z_{i-1}^p = j | g; X^{(t)}, Y^{(t)}, \Pi^{(t)}, \Omega^{(t)}) \end{aligned}$$

We use Newton's method to perform the maximization step. The gradient for Q function in (5) can be computed as follows

$$\frac{\partial Q}{\partial \pi_j} = \sum_{ij} \frac{D_{ij}}{\pi_j} + \frac{E_{ij}\tau_i}{1 - \tau_i(1 - \pi_j)}$$

The Hessian matrix for the Q function in in (2) can be computed as follows

$$\frac{\partial^2 Q}{\partial \pi_j^2} = \sum_{ij} -\frac{D_{ij}}{\pi_j^2} - \frac{E_{ij}\tau_i^2}{(1 - \tau_i(1 - \pi_j))^2}$$

Similarly, the derivation of Q function in Ω can be done by replacing all Z^p variables with Z^m.
