# Supplementary figures and images for: Spatial Localization of Recent Ancestors for Admixed Individuals
Source: G3 (Bethesda). 2014 Nov 3;4(12):2505–18. doi: 10.1534/g3.114.014274 (PMC4267945; doi:10.1534/g3.114.014274)

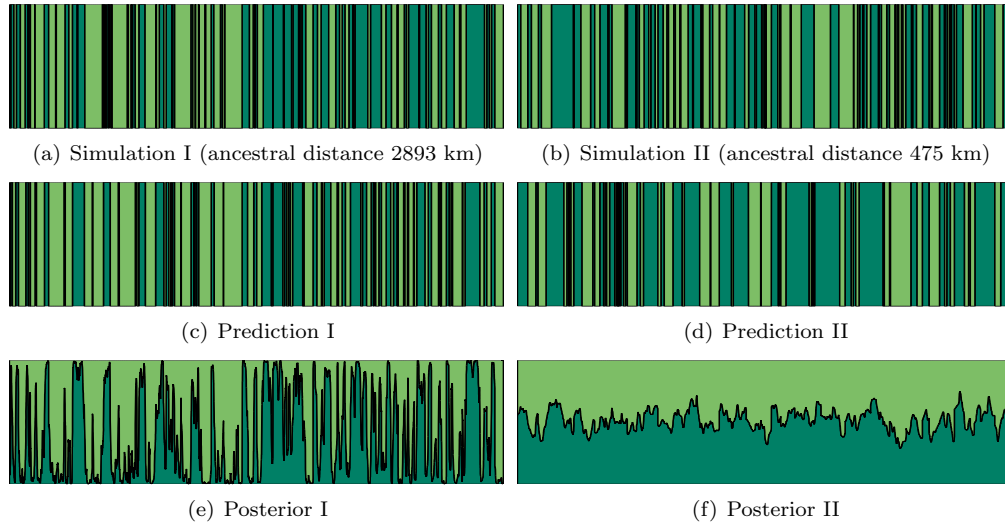

Figure S1: Example of local ancestry prediction results for distant and close ancestors.

Supplement: Supporting Information [file supp_g3.114.014274_FigureS1.pdf]

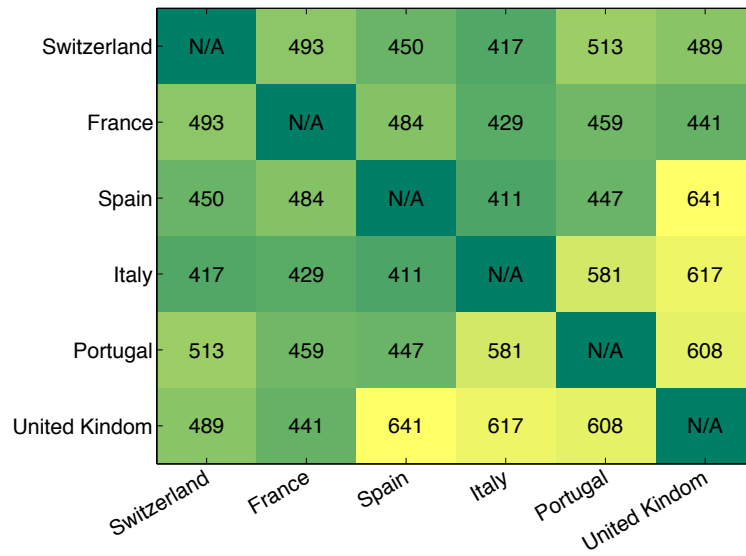

Figure S2: Average Prediction error (Km) for six country pairs with largest populations.

Supplement: Supporting Information [file supp_g3.114.014274_FigureS2.pdf]
