# Supplementary material for: Spatial Localization of Recent Ancestors for Admixed Individuals
Source: G3 (Bethesda). 2014 Nov 3;4(12):2505–18. doi: 10.1534/g3.114.014274 (PMC4267945; doi:10.1534/g3.114.014274)
Supplement: Supporting Information [file supp_g3.114.014274_TableS2.pdf]

Table S2: Average distance between inferred and true ancestry locations in simulated admixed individuals from POPRES data. Independent SNP model denotes the extension of SPA that ignores admixture-LD. It can also be understood as SPAMIX with completely random transition probability between nearby SNPs. SPAMIX (logistic) represents simulation results starting from haplotypes generated at a location on a map using a Bernoulli sampling from the logistic gradients (see Methods). Parenthesis denote the standard deviations. We found that Linkage Disequilibrium (LD) significantly affects the ancestry inference as well as the local ancestry inference in unaccounted for. We observe more recombination events than expected if using the correct recombination probability (used in simulations). We circumvent this bias multiplying the transition probability by a factor 10^{-1}, 10^{-2}, 10^{-4} and 10^{-5} for the pruned SNP list with 0.1, 0.2, 0.5 and 0.8 pruning thresholds. 44,699, 72,418, 136,284, 194,432 SNPs were retained at the 4 pruning thresholds.

No. of generation		2	4	6	8
Independent SNP model	Pruned SNP (0.1)	995(550)	961(540)	974(539)	987(537)
	Pruned SNP (0.2)	899(487)	880(491)	864(466)	927(491)
	Pruned SNP (0.5)	809(444)	823(448)	819(436)	837(444)
	Pruned SNP (0.8)	834(441)	810(429)	812(442)	799(447)
SPAMIX	Pruned SNP (0.1)	549(318)	558(314)	567(334)	546(326)
	Pruned SNP (0.2)	548(329)	550(326)	541(295)	562(336)
	Pruned SNP (0.5)	551(390)	557(359)	590(371)	588(467)
	Pruned SNP (0.8)	580(478)	589(557)	634(576)	586(538)
