# Supplementary material for: Spatial Localization of Recent Ancestors for Admixed Individuals
Source: G3 (Bethesda). 2014 Nov 3;4(12):2505–18. doi: 10.1534/g3.114.014274 (PMC4267945; doi:10.1534/g3.114.014274)
Supplement: Supporting Information [file supp_g3.114.014274_TableS3.pdf]

Table S3: The outliers from SPAMIX analysis. They are POPRES admixed individuals with ancestral predictions inconsistent with their self-reported ancestries.

POPRES ID	self	father	PGF	PGM	mother	MGF	MGM	Pred. Locations
4183	Austria	Austria	Austria	Czech Republic	Poland	Russia	Switzerland	(52.26 12.61),(43.78 -9.81),(41.63 16.61),(52.44 13.30)
28710	France	Poland	Germany	Poland	France	France	France	(39.47 5.82),(37.97 14.65),(40.99 15.31)
24943	Sweden	Sweden	Sweden	Sweden	Finland	Russia	France	(48.03 6.91),(48.09 6.67),(57.35 8.79)
20086	France	France	France	France	France	Switzerland	France	(35.62 13.04),(43.65 16.14)
5550	Germany	Switzerland	Switzerland	Switzerland	Germany	Germany	Germany	(46.45 23.86),(55.66 -7.40)
47799	Germany	Germany	Russia	Germany	Germany	Germany	Germany	(50.20 14.77),(53.02 3.44)
32002	France	France	France	France	Turkey	Turkey	France	(35.85 10.05),(40.75 13.59)
27995	Poland	Poland	Poland	Poland	Russia	Russia	Poland	(55.65 12.49),(47.67 10.56)
38489	Russia	Russia	Germany	Germany	Switzerland	Switzerland	Russia	(46.59 5.62),(46.67 7.38),(49.86 -0.22)
7251	Austria	Switzerland	Switzerland	Switzerland	Austria	Austria	Austria	(38.64 -6.89),(62.19 30.99)
17323	Switzerland	Russia	Russia	Russia	Switzerland	Switzerland	Switzerland	(50.99 -1.41),(46.35 10.78)
20046	France	Russia	Russia	Russia	Poland	Poland	Poland	(37.25 14.09),(44.40 9.91)
24429	France	Russia	Russia	Russia	France	France	France	(50.59 1.93),(44.02 4.97)
39106	Israel	Greece	Greece	Greece	Russia	Germany	Sweden	(41.68 4.76),(36.35 23.47),(41.78 5.04)
49793	France	Romania	Romania	Romania	Russia	Russia	Russia	(41.31 9.54),(40.83 19.25)
47137	France	France	Germany	Germany	Austria	Switzerland	Switzerland	(38.01 12.40),(41.96 12.49)
34848	Egypt	Turkey	Turkey	Turkey	France	France	France	(38.75 9.47),(37.47 14.32)
10635	France	France	France	France	Russia	Russia	Bulgaria	(53.28 5.54),(45.50 5.56),(50.11 7.61)
18548	Czech Republic	Germany	Germany	Germany	Russia	Russia	Russia	(42.54 13.24),(38.67 9.20)
22423	Russia	Ukraine	Ukraine	Ukraine	Russia	Russia	Russia	(53.32 7.55),(50.79 17.17)
13411	France	Russia	Russia	Russia	France	France	France	(39.22 9.31),(46.77 5.19)
42867	Switzerland	Switzerland	Switzerland	Russia	Switzerland	Switzerland	Switzerland	(47.42 16.49),(49.47 -4.12)
33744	Switzerland	Switzerland	Russia	Germany	Spain	Switzerland	Switzerland	(42.39 13.98),(40.81 5.44),(50.07 2.19)
31350	Israel	Romania	Romania	Romania	Russia	Russia	Russia	(42.23 7.63),(38.30 19.55)
15990	France	Russia	Russia	Russia	Greece	Greece	Greece	(38.60 5.52),(38.88 15.46)
